# Availability of Spanish-Language Medical Apps in Google Play and the App Store: Retrospective Descriptive Analysis Using Google Tools

**DOI:** 10.2196/17139

**Published:** 2020-12-03

**Authors:** Inmaculada Grau-Corral, Pau Gascon, Francisco J Grajales III, Belchin Kostov, Antoni Sisó Almirall

**Affiliations:** 1 Fundación iSYS Barcelona Spain; 2 Hospital Clínic Barcelona Spain; 3 Centre for Social Innovation & Impact Investing, Sauder School of Business, University of British Columbia Vancouver, BC Canada; 4 Primary Healthcare Transversal Research Group, Institut d’Investigacions Biomèdiques August Pi i Sunyer (IDIBAPS) Barcelona Spain; 5 Consorci d'Atenció Primària de Salut Barcelona Esquerre (CAPSBE) Barcelona Spain; 6 University of Barcelona Barcelona Spain

**Keywords:** apps, mobile health, mHealth assessment, evaluation studies, health apps, ratings, mobile apps, Spanish apps, patient apps, Google Advanced Search, mobile phone

## Abstract

**Background:**

The number of medical and health apps in the App Store and Google Play repositories has been increasing in the recent years, and most of these apps are in English. However, little is known about the domain of Spanish health apps and their evolution.

**Objective:**

The aim of this study was to perform a retrospective descriptive analysis of medical apps for patients in the Spanish language by using Google search tools over a 5-year period and to compare the results by using a reproducible methodology to obtain a better knowledge of the medical apps available in the Spanish Language.

**Methods:**

Over a 5-year period, medical apps were catalogued using a Google-based methodology. Keywords of the first 14 categories of the International Classification of Diseases, Tenth Revision, were selected, and in December of each year, searches of the URLs of Google Play and the App Store were conducted using Google Advanced Search. The first 10 results were taken, and apps meeting the inclusion criteria were selected and rated with the iSYScore method.

**Results:**

Out of a sample of 1358 apps, 136 met the inclusion criteria. The 3 main categories of the medical apps were in the fields of endocrinology (diabetes), respiratory (chronic obstructive pulmonary disease, asthma, and allergies), and neurology (multiple sclerosis, Parkinson disease, and Alzheimer disease). Few apps were maintained over the 5 years. Only 10 of the 136 apps were maintained for 3 years or more. There was a large number of original apps in other languages that were translated into Spanish (56/136, 41.2%). In the last year of the study, the main reason (73/280, 26.1%) for discarding an app was the date of the last update.

**Conclusions:**

The market of Spanish apps is poor; only few apps have appeared repeatedly over 5 years. Differences were found with the international market in terms of apps related to mental health, heart and circulatory system, and cancer, and coincidences were found in the relevance of apps for diabetes control.

## Introduction

### Background

At its early stage of development, access to smartphone apps is limited to specific provider-customer relationships and the number of smartphone users is less. However, the smartphone market has accelerated over time, reaching 86% of the population in Europe [1]. Indeed, since the launch of mobile app platforms in 2008, including Google Play and App Store (for Android and iPhone operating system [iOS], respectively), the number of apps available has increased [2,3]. In the Apps store market, it is estimated that about 325,000 apps are dedicated to health [4].

The level of complexity associated with mobile health (mHealth) app products is heterogeneous, for example, a simple app that promotes a charity is not the same as a more complex one with Food and Drug Administration or European Union accreditation, which allows the monitoring of a patient with a heart disease. Likewise, there is also heterogeneity in the type of disease that is covered. For example, there are a large number of apps for monitoring patients with diabetes and a much lesser number of apps for patients who have undergone a transplant [4]. Moreover, there is also a cultural gap; most studies are conducted with apps that are developed in English. The inclusion criterion of most of the studies analyzing apps is that they were in English. There are only few studies referring to apps only in Spanish such as clinical trials, literature reviews, or meta-analyses. A simple search in PubMed for this type of studies with the terms “App” and “Spanish” yielded 23 results, while the search for “English” and “App” returned 997 results at the end of 2019.

Interventions aimed at Spanish cultural adaptation of an original development in English are more frequent. While there are official sites in Spain with rigorous selection criteria involving a small number of apps [5-7], multilingual platforms with a much larger number of apps such as the ORCHA Health App Library [8], Healthy living apps of Vic Health Foundation [9], and My Health Apps [10] dominate the market.

Regarding the use of mHealth in health care, there are interesting studies on the opportunities offered by its implementation for a safe and effective deployment in Latin America [11,12]. They describe the need to implement a regulation that guarantees the safety of developments [13,14], the protection of personal data [15], and all ethical aspects such as the European Union's “medical device” regulation [16]. However, the incidence and implementation of mHealth in the health system is not the focus of this study.

The objective of this study was to analyze the proposals that Spanish-speaking citizens can find in 2 large app stores, that is, Google Play and App Store, in Spanish. Users currently access the apps through 2 approaches: by recommendation or by filter. The “recommendation” can come from the health professional, a government, or public health authorities. The “filter” approach is conditioned by the results of the search engines of companies such as Google or Apple. Studies on policies and regulations set their point of view on access to apps by “recommendation.” In this study, we focused on the access to patient apps by “filter;” specifically, we used the “Google Advanced Search” tool.

### Prior Work

In 2014, a research group of the iSYS Foundation developed the iSYScore [17] with the objective of obtaining indicators to help patients choose health apps. This score is similar to a triage scale, which allows agile selection and recommendations, but is not an accreditation. It does not delve into elements such as security that accreditations give nor in the efficiency of the app as the regulations of the Food and Drug Administration or European Union do. Once the iSYScore scale was ready, it was applied to a sample of apps obtained on the best results shown by Google Advanced Search in various groups of diseases. These annual collections have been recognized by the European mHealth Hub [18]. Since 2014, the iSYS Foundation has been collecting about 280 apps every year with Google Advanced Search, which verifies that they have met all the inclusion criteria and have applied the iSYScore in order to recommend a sample of the best results. However, the total number of apps analyzed annually is higher than that obtained by the methodology used by our group because recommendations of patient associations and registrations are also collected. Therefore, in order to achieve homogeneous results, only apps obtained with Google tools were included in this study.

### Objectives

The aims of this study were (1) to analyze the findings obtained with a sample of medical apps per group of disease for 5 years that were selected by Google Advanced Search, (2) to describe the evolution of Spanish health apps over a 5-year period, and (3) to compare the health app studies available in Spanish with those in English or other languages.

## Methods

### Methodological Framework

The methodology in this study has 2 sections. The first section explains how the apps included in the sample were extracted and selected. The second section explains how the analysis on the selected apps was conducted.

#### Extraction and Selection of Apps

Every year, since 2014, researchers from the iSYS Foundation collected a sample of apps in Spanish for analysis. The analysis of this work corresponds to the samples obtained in 2014, 2015, 2016, 2017, and 2018.

To collect apps, researchers used the following methodology.

1. Selection of keywords by a set of diseases defined in the International Classification of Diseases, Tenth Revision (ICD-10) [19]: In the first year, the keywords selected were the most frequently used in the ICD-10 text using a word counter. In the following year, the researchers added words from websites belonging to patient associations. In the following years, the same set of keywords was used, with some small variation at the discretion of the researchers.
2. After selecting the keywords, the researchers used Google Advanced Search to obtain apps. For each set of diseases (for example, “Infectious and parasitic illnesses”), researchers put the keywords separated by spaces (OR), chose the Spanish language option, and entered the URL of Google Play for the first search and the one of Apple Store for the second one. The first 10 results of each platform were selected.
3. By having 14 groups of ICD-10 diseases with their keywords and targeting the search of 2 platforms, the expected result was 14×10×2=280 apps to analyze each year.

For the selection of apps, the following inclusion criteria were established: (1) Spanish language; (2) adequacy: e-books and podcasts were not accepted, as well as apps where cancer was a horoscope sign and apps with the absence of medical device accreditation if it was an app for medical diagnosis, treatment, or monitoring; (3) targeted audience: not for professionals; (4) availability: no password or geographic filter or other problems of availability; and (5) exceed a cut-off score according to the iSYScore (11 points out of a maximum of 48). In 2016, 2 new criteria were added: (1) to have a minimum number of downloads (N≥500) and (2) to have had an update the year before the sample was captured.

The exclusion criteria were duplicated apps and apps that promised miracle cures. The total sample of apps to be analyzed that met the inclusion criteria would be presented by ICD-10 disease groups in time series (one per year) to observe trends.

#### Analysis of the Selected Apps

To analyze evolution, the researchers considered the following parameters:

1. The number of apps per group of diseases in the sample during the study period and how many apps appeared recurrently over the years and which ones did not.
2. The main reason for discarding per year, given the observation of changes that have occurred during the capture of apps, was obtaining many results in English, despite the language filter.
3. Durability of the analyzed apps: In 2019, the researchers investigated the apps recommended in the previous years to observe their evolution.
4. Finally, the researchers investigated the possible factors influencing the durability of the apps depending on whether they were in native Spanish or translated or whether it depended on the promoter of the app.

Details to make these 4 sections:

1. The researchers would complete a table with the apps selected each year and group them by group of diseases and year. The table would allow them to obtain the totals and to keep track of those apps that appeared for more than 1 year.
2. To collect the main reasons for discard by year, the researchers had to add in tables the reason for discard by platform (Google Play and App Store) and by year, and detect if the main reason for the change varied.
3. To analyze the durability of the apps, the researchers searched the most established categories. For the analysis of the stability of the developments by disease group, a proxy variable was established and defined as a ratio: the relation between the number of repeated appearances by the number of individual apps of the category during the study period. The researchers considered that for a more “stable” disease group, apps would appear for more years in the annual sample. For example, if a disease group only has an app, and this app appears 5 years in a row, the ratio would be 5 (maximum value), and if it only appears 1 year, the ratio would be 1 (minimum value). Additionally, in 2019, apps from previous years would be revalidated to determine if they continue to meet the inclusion requirements or have disappeared. This will allow one to observe those that are no longer available. This will also allow us to observe which groups of diseases have a higher rate of “disappearance.” To select the most consolidated categories, it was agreed to select those apps with a stability ratio higher than the average and a percentage of disappearance lower than the average. To observe the most unstable categories, the criterion used was those apps that had a ratio lower than the average and a percentage of disappearance higher than the average.
4. To study the possible factors influencing the durability of the apps, the researchers explored the developers’ localization whether they were native apps in Spanish or translated from other languages. In addition, the promoter of the initiative was noted, in case this factor had an influence on durability.

Regarding the promoter of the apps, 6 categories were established: (1) health professionals that included individuals, health providers, and universities; (2) companies (neither pharmaceutical industry nor start-up); (3) nonprofit organizations, including foundations, associations, and scientific societies; (4) pharmaceutical industry; (5) patients and patient associations; and (6) projects and start-ups.

### Statistical Analysis

The analysis of the potency and statistical significance used in studies that test a hypothesis does not apply to retrospective descriptive studies such as this one. Therefore, the results were summarized using descriptive statistical techniques such as percentages and means.

## Results

### Extraction and Selection of Apps

A sample of apps was collected over a 5-year period; 1358 apps (annual average 271.6, mean 263.5) were found using the described methodology. Every year, there was a significant decrease in the inclusion of the number of apps as these apps did not meet the inclusion criteria: of the 1358 obtained by our methodology, only 210 met the inclusion criteria (Table 1).

**Table 1:** Details of the number of apps collected over the years in this study.

Description	Year 2014	Year 2015	Year 2016	Year 2017	Year 2018
Apps collected (n=1377)	247	280	280	271	280
Apps that met inclusion criteria (n=210)	26	55	42	48	39

**Figure 1:**
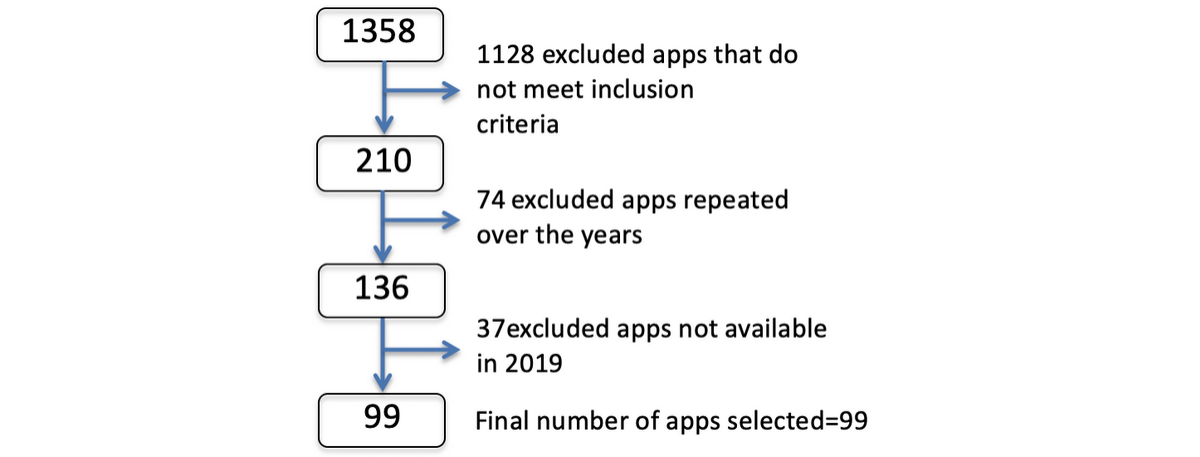
Flowchart of the selection of apps.

The searches by disease groups did not reach, in some cases, the 20 results expected in the methodology. Reviewing the 210 outcomes that met the inclusion criteria over 5 years, 74 repetitions of apps were found over the years and extracted, leaving a total of 99 different apps (Figure 1).

### Analysis of the Selected Apps

During the study period, in which each group of diseases could have up to 20 different apps per year, a maximum of 8 and a minimum of 0 were found (Table 2).

**Table 2:** Evolution of the health apps included each year, classified by 14 ICD-10^a^ disease groups (n=210).

Apps as per ICD-10	Number of apps in each year				
	Year 2014 (n=26)	Year 2015 (n=55)	Year 2016 (n=42)	Year 2017 (n=48)	Year 2018 (n=39)
Infectious and parasitic diseases (n=16)	1	6	3	2	4
Cancer (n=21)	4	8	5	2	2
Blood diseases (n=8)	1	2	2	1	2
Endocrine, nutritional and metabolic diseases (n=32)	5	6	5	11	5
Mental and behavioral disorders (n=8)	1	2	2	2	1
Diseases of the nervous system (n=28)	4	8	5	5	6
Eye diseases (n=11)	1	4	2	2	2
Ear diseases (n=12)	0	4	2	3	3
Circulatory system diseases (n=12)	2	3	2	3	2
Diseases of the respiratory system (n=18)	—^b^	3	3	6	6
Diseases of the digestive system (n=12)	1	3	3	3	2
Skin diseases (n=11)	3	2	3	3	0
Diseases of the musculoskeletal system (n=12)	3	2	2	3	2
Genitourinary system diseases (n=9)	0	2	3	2	2

^a^ICD-10: International Classification of Diseases, Tenth Revision.

^b^Not available.

Most apps were associated with the endocrine and nervous systems and cancer-related diseases. Apps dedicated to diabetes were included under endocrine diseases, and these apps were predominant over apps dedicated to obesity. With regard to diseases of the nervous system, tracking apps for multiple sclerosis and Parkinson disease, symptoms of meningitis, or information for relatives of people with Alzheimer disease were selected. With respect to cancer, the majority of the apps obtained by Google Advanced Search were related to skin cancer and the follow-up of skin lesions. There were also results involving apps providing information about breast cancer. In 2014, no data were collected in the “Diseases of the respiratory system.” Despite this fact, this domain was in the fourth place in terms of total number of apps. Pollen alerts, asthma, and chronic obstructive pulmonary disease follow-up were the themes of the apps in the diseases of the respiratory system domain.

### Reasons for Excluding the Apps

The main reason for the exclusion of apps was that they were not in Spanish (323/1358, 23.8%), although the language filter was activated in the search (Table 3).

**Table 3:** Reasons for app exclusion.^a^

Reasons, operating systems		Number of apps in each year				
		Year 2014	Year 2015	Year 2016	Year 2017	Year 2018
**It was not in Spanish (n=323)**						
	Android (n=182)	36	64	15	40	27
	iOS^b^ (n=141)	5	51	43	24	18
	Total	41	115	58	64	45
**It was for health professionals (n=231)**						
	Android (n=114)	36	20	47	7	4
	iOS (n=117)	54	37	16	7	3
	Total	90	57	63	14	7
**It was not suitable (n=227)**						
	Android (n=106)	54	11	22	10	9
	iOS (n=121)	30	19	28	15	29
	Total	84	30	50	25	38
**Not available (n=156)**						
	Android (n=80)	54	7	6	2	11
	iOS (n=76)	56	3	10	2	5
	Total	110	10	16	4	16
**Repetitions (n=114)**						
	Android (n=37)	0	1	2	5	29
	iOS (n=14)	0	1	4	4	5
	Both (n=63)	2	6	22	12	21
	Total	2	8	28	21	55
**Last update date (minimum the year prior to the annual collection) (n=152)**						
	Android (n=88)	—^c^	—	21	36	31
	iOS (n=64)	—	—	2	20	42
	Total	—	—	23	56	73
**Downloads <500 (n=84)**						
	Android (n=76)	—	—	19	33	24
	iOS (n=8)	—	—	0	6	2
	Total	—	—	19	39	26
**Did not rate 11 points on the iSYScore (n=127)**						
	Android (n=57)	13	23	12	5	4
	iOS (n=70)	22	24	6	11	7
	Total	35	47	18	16	11

^a^There may be more than one reason for exclusion of apps.

^b^iOS: iPhone operating system.

^c^Not available.

A significant number of apps (231/1358, 17.0%) were discarded because they were aimed at health professionals mostly in the genitourinary and musculoskeletal disease fields. Adding the number of downloads (minimum 500) and the last update date (minimum the year prior to the annual collection) in 2016 led to an increase in discard rates in successive years. In parallel, the number of discards decreased due to not reaching the minimum score with the iSYScore.

### Stability of the Results Over Time

On revalidation of the apps from previous years in 2019, it was found that 37 out of 136 apps (27.2%) were no longer available (Table 4). Only 10 apps appeared 3 or more times over the 5-year period.

**Table 4:** Stability of the results through the years.

Number of apps	Year 2014	Year 2015	Year 2016	Year 2017	Year 2018	Total	Repeated apps	Apps without repetition
Annual results (n)	26	55	42	48	39	210	74	136
Apps disappeared in 2019, n (%)	12 (46)	18 (33)	10 (24)	4 (8)	0 (0)	44 (20.9)	7 (10)	37 (27.2)
Apps remaining in 2019 (n)	14	37	32	44	39	166	N/A^a^	99

^a^N/A: Not applicable.

The apps most repeated over the 5-year study period were those related to the genitourinary diseases, whereas the least repeated were those associated with eye diseases. The most consolidated categories were those with a higher than average ratio and a lower than average percentage of disappearance and included the following 3 categories: endocrine, nutritional and metabolic diseases, genitourinary diseases, and infectious and parasitic diseases (mainly HIV infection). Three categories were the most unstable, having a lower than average ratio and a higher than average disappearance percentage: diseases of the digestive system, cancer, and mental and behavioral disorders (Table 5).

**Table 5:** Stability of the results through the years by group of disease (n=210).

ICD-10^a^ group	Repeated apps (n=74)	Unique apps without repetition (n=136)	Stability ratio	Disappeared apps, n (%)	Apps in 2019 (n=99)
Infectious and parasitic diseases (n=16)	6	10	1.6	2 (20)	8
Cancer (n=21)	8	13	1.6	7 (54)	6
Blood diseases (n=8)	3	5	1.6	2 (40)	3
Endocrine, nutritional and metabolic diseases (n=32)	16	16	2.0	2 (13)	14
Mental and behavioral disorders (n=8)	1	7	1.1	3 (43)	4
Diseases of the nervous system (n=28)	13	15	1.9	5 (33)	10
Eye diseases (n=11)	2	9	1.2	0 (0)	9
Ear diseases (n=12)	1	11	1.1	2 (18)	9
Circulatory system diseases (n=12)	3	9	1.3	4 (44)	5
Diseases of the respiratory system (n=18)	6	12	1.5	2 (17)	10
Diseases of the digestive system (n=12)	4	8	1.5	5 (63)	3
Skin diseases (n=11)	3	8	1.4	1 (13)	7
Diseases of the musculoskeletal system (n=12)	3	9	1.3	1 (11)	8
Genitourinary system diseases (n=9)	5	4	2.3	1 (25)	3

^a^ICD-10: International Classification of Diseases, Tenth Revision.

### Possible Factors Influencing the Durability of the Apps

Most of the apps found were originally developed in Spanish and were from Spain and Latin America (80/136, 58.8%). However, those that had been translated from another language showed a tendency to be more durable (Table 6).

**Table 6:** Native Spanish apps and apps that were translated from other languages to Spanish (n=136).

Language type of apps, availability		Values, n (%)	
**Spanish apps**			
	Available		55 (69)
	Not available		25 (31)
	Subtotal		80 (58.8)
**Apps translated from other languages**			
	Available		44 (79)
	Not available		12 (21)
	Subtotal		56 (41.2)

### Apps According to Promoter

The most stable app developments (with less disappearance of products) were those in the Company category (Table 7), while the most spurious ones were those related to projects and start-ups, as well as those led by the pharmaceutical industry.

**Table 7:** Apps selected and classified by promoter (n=136).

Promoter type and availability		Values, n (%)
**Health care professionals**		
	Available	7 (70)
	Not available	3 (30)
	Subtotal	10 (7.3)
**Company**		
	Available	36 (82)
	Not available	8 (18)
	Subtotal	44 (32.3)
**Pharmaceutical industry**		
	Available	7 (64)
	Not available	4 (36)
	Subtotal	11 (8.1)
**Nonprofit public foundation**		
	Available	25 (69)
	Not available	11 (31)
	Subtotal	36 (26.5)
**Patients**		
	Available	7 (70)
	Not available	3 (30)
	Subtotal	10 (7.3)
**Start-up+project**		
	Available	15 (60)
	Not available	10 (40)
	Subtotal	25 (18.4)

## Discussion

### Principal Findings

The search for the best health apps in the Spanish language using the Google Advanced Search algorithm resulted in only 136 apps meeting the inclusion criteria. Of these, a significant proportion (56/136, 41.2%) corresponded to English translations and a significant percentage disappeared over the 5-year study period. Only 10 apps appeared for 3 or more years (the most stable). Apps dedicated to diabetes were the most common [15] and had frequent medical device accreditation. A recent meta-analysis on the use of mHealth to support patients with diabetes showed positive results [20]. Despite the number of apps found in neurology (Alzheimer disease, epilepsy, multiple sclerosis, etc), there were only few studies on the effectiveness of those apps [21].

In the cancer section, the keyword “cancer” had to be discarded since 2014, as it offered better-positioned results related to horoscopes. Results related to possible nonevidence-based cures were also ruled out. According to the iSYScore results, the best positioned app was the Spanish translation of the ASCO App Cancer.net [22]. There is a lack of patient follow-up apps besides those related to skin or breast cancer, which have been shown to be useful in some studies [23-26]. One possible explanation may be that these apps are not available on commercial platforms (Google Play or App Store). Reviewing reasons for the apps that were discarded, researchers found that the main reason was that apps selected were not in Spanish (323/1358, 23.8%), despite Spanish language being selected in Google Advanced Search.

Indeed, in 2014, the “Spanish language” was added as a keyword in order to obtain more adequate results. The tendency to present results in other languages by these platforms has decreased over time (Table 3). Cancer, skin, and digestive system diseases were the most affected by this problem. Regarding discarded apps because of inadequacy (Table 3), apps related to mental and behavioral diseases showed the most interesting results. These had nonvalidated treatments more frequently (eg, cure of schizophrenia with phone vibrations) or were not available to the public because they were included as part of a controlled study for which an individual had to be included in the study in order to have access to the app. Other results not related to health (singers or horoscope signs) were also found in other categories.

### Comparison With Studies in Other Languages

We compared our results with those of other studies such as the report by the American IQVIA, “The Growing Value of Digital Health” [4] and the German Research2Guidance “mHealth Economics 2017-Current Status and Future Trends in Mobile Health” [27]. IQVIA performed an analysis on the number of apps available by category. The category of “Health condition management” and specifically under the section “Disease Specific” showed a number in which the apps dedicated to mental disorders, diabetes, and the heart and circulatory system dominated the top positions in terms of the number of apps. The same report suggested that the evidence of the effectiveness of the apps described in 571 published studies in 5 patient populations on reductions in the utilization of acute care: prevention of diabetes, diabetes, asthma, cardiac rehabilitation, and pulmonary rehabilitation.

With regard to Research2Guidance, we found that comparison of our results was not adequate since their work was based on expert surveys while our results were based on searches using Google tools. That study reported that the most attractive app development fields are related to physicians (30%), diabetes (27%), the heart, blood, and circulatory system (24%), medications (24%), healthy lifestyles (22%), hospital efficiency (19%), and mental health (17%). Both reports coincide in the categories of diabetes, heart problems, and mental health as those that aroused most interest, although in different order of priority.

In this study, a large number of apps were found related to diabetes, cancer, and diseases of the nervous system. The apps found to be the most stable over time were those belonging to the categories of diabetes, infectious diseases, and kidney diseases, thereby disagreeing with the previously mentioned studies with the exception of the main category, that is, diabetes.

### Analyzing the Evolution

The number of apps discarded because the Google search showed results in English and other languages significantly decreased in the last year of the study. However, repetitions in the same category and platform increased the number of discards in the same year. Small changes to the URL of the repeated apps might explain this fact. Over the years, there was a trend toward an increase in the number of apps ruled out due to lack of updates. However, the number of apps ruled out by a low iSYScore decreased over time, indicating a higher sample quality that exceeds the inclusion criteria. Regarding the different categories, the robustness of the endocrine (mostly diabetes) and nervous system categories over time was of note, while apps dedicated to cancer decreased.

### Limitations of This Study

An obvious limitation of this study was the dependence on the results of the Google algorithm for the selection of the most representative results. This results in a volatile and dependent return. The decrease in the results on cancer and the digestive system and mental health disorders suggests that developments occur outside major markets such as in research fields [28] or payment software.

## Abbreviations

ICD-10: International Classification of Diseases, Tenth Revision
iOS: iPhone operating system
mHealth: mobile health
